# Report on the Coucours Relative to the Question of Apparent Death, and of the Means of Preventing Premature Burials

**Published:** 1849-01

**Authors:** James Bryan

**Affiliations:** member of the National Medical Association, Professor of Surgery in Geneva Medical College


ART. II.—*Report on the Coucours relative to the Question of Apparent Death, and of the means of preventing Premature Burials.* [Commission, Messrs. Dumeril, Andral, Magendie, Serres, Rayer—reporter.]

From the "Comptes Rendus Hebdomadaires des Seances de l'Academié des Sciences." Translated by JAMES BRYAN, M. D., member of the National Medical Association, Professor of Surgery in Geneva Medical College, N. Y.

The very old opinion of the uncertainty of the signs of death, strengthened unfortunately by the deplorable errors of ignorance and haste, has been increasing in France for about a century, since the publication of the celebrated dissertation of Winslow: An mortis incertae signa minus incerta a chirurgicis quam ab aliis experimentis? Paris, 1740. A French translation of this little work, published by Bruhier d'Ablincourt, under the title of, "Dissertation sur l'incertitude des signes de la mort," with the remarks of the same author on the necessity of a general regulation on the subject of interment and embalming, produced a strong sensation in the public mind.

Bruhier collected a hundred and twenty two cases, whose title alone was enough to produce alarm, viz:

"Fifteen cases of persons buried alive; Four cases of persons opened before their death; one hundred and three cases of persons supposed dead, who were not, the greater number of whom were restored to life, previous to inhumation."

Bruhier was so careless as not to be critical in the choice and appreciation of the facts which composed this long enumeration. Louis, secretary of the Royal Academy of Surgery, thought to allay the excited public feeling. He demonstrated with a good deal of ingenuity, that a large number of premature burials, cited by Bruhiér, were not well substantiated, and that the greater portion of the real and well established cases were due to the deplorable ignorance of those who were connected with them. The letters of Louis "on the certainty of the signs of death," effected the object completely. He may be blamed for considering certain phenomena, such as the insensibility of the globe of the eye to the touch, the formation of a glairy veil on this organ, as certain signs of death; but he has the credit of having proved, from observation made on more than a hundred subjects, dead of various diseases, that *cadaveric rigidity* is a certain sign of death.

Nyston afterwards confirmed this by numerous examples.

Stimulated by the experiments of Haller on irritability, he showed that the absence of muscular contractility, under the influence of galvanic agents or of direct stimulants, was a new and certain sign of death; and one which could be verified before general putrefaction of the body. The experiments of Nyston were made (in 1811) in the Hopital de la Charité, on about forty subjects, which had died of the most common diseases.

Since this, it has been generally admitted, at least in France, that death may easily be determined, by physicians, before decomposition of the body. Nevertheless, a small number of them still insist that putrefaction is the only certain sign of death.*

For the establishment of this opinion, the names of foreign physicians of distinction are quoted. The anxiety of the public has also been increased by the foundation, in a number of towns in Germany, of dead houses, destined to receive the bodies of persons, until putrefaction shall have commenced.

Such was the opinion and state of the question when, at the session of February 13th, 1837, a letter was read to the Academy of Sciences, from Manni, professor in the University at Rome, in which he proposed to advance funds for a special prize of 1500 francs, for the best memoir on the question of apparent deaths, and the means of remedying the serious accidents which too frequently result from them. After having consulted the Section on Medicine and Surgery, the Academy took the offer of Manni into consideration. A royal ordinance, dated August 5th, of the same year, authorized the acceptance of the funds, and their appropriation to the object proposed. Consequently, the Academy proposed, in 1837, for a prize to be awarded in 1839, the following questions:

What are the distinctive characters of apparent death? What are the means of preventing premature burials?

The Academy [received seven manuscript memoirs. Several of them confirmed important opinions which had not before been established. The awarding of the prize was postponed to the year 1842.

The Academy hoped, by this new delay, that the authors would be able to give their productions that degree of perfection which the importance of the subject demanded. In 1842, the Academy received again, seven memoirs. The commission, disappointed in their aims, decided, with regret, that this year, also, no prize could be awarded.

---

NOTE.—The citizens of Philadelphia will long remember the great caution taken by the late Dr. Physic in his last testament, that decomposition should take place previous to his burial. For this purpose his body, at his own request, was kept in a warm room for several days, until it became offensive to the whole house.

In submitting this subject to a concours, for the year 1846, and for the third time, the commission feel bound to declare that the reports of premature interments, indicate, generally, rather the ignorance and carelessness of the authors of these evils, than the uncertainty of science. It demands, with other things, a complete exposition of what is actually known on the question proposed, in addition to which, it desires, especially, new observations on the condition of life and death in order to render the diagnosis more prompt and sure in the small number of cases which are doubtful in the mind of the physician.

Six memoirs have been addressed to the commission for this new concours. The Academy have sent to the commission on the prize of Manni, several printed works, and some other documents, relative to the question proposed.

Only one memoir has appeared to the commission worthy of reward.

The first question proposed by the Academy was the following:

*What are the Characteristics of Apparent Deaths.* Before entering upon the first question, the author of the memoir observes that the history of apparent deaths, show more than any other perhaps, how necessary it is to be cautious in adopting an observation, and understanding fully the importance and truth of the fact given.

With numerous observations on premature interments, cited to prove the uncertainty of the science in reference to the signs of death, the author of the memoir, Dr. Bouchut, thinks that all these facts should be carefully studied and sifted in proportion to their importance. The author has shewn in the matter a talent for criticism, of which the celebrated Louis has given a model, which it is difficult to surpass. Dr. Bouchut classes the observations on premature interment in three categories.

The first of these confirms the mistakes made by physicians. One of these deplorable errors has been attributed to Vesalius. According to some this celebrated anatomist applied the scalpel to the body of a Spanish gentleman still living; according to others a female was the victim of the homicidal error. Two versions so different render the fact doubtful. Dr. Bouchut remarks, that no contemporary Spanish writer makes mention of the error attributed to Vesalius. Dr. Fernandez Morejon, author of a bibliographical history of Spanish medicine, and Professor Burgrave, of Brussels, who was devoted to learned researches on the life of Vesalius, have equally asserted that this pretended error of Vesalius was a fable, invented by calumny, a fable unfortunately believed for a long time, and promulgated by Albinus and by Boerhaave in a preface to the works of the celebrated Belgian anatomist.

The second mistake was committed by Pere, a celebrated accoucheur. A female at full term appeared entirely dead. Called to perform the Cæsarean operation, Pere reports, that the assistants, persuaded that the female was entirely dead, pressed him to operate. "I also thought so, for I observed no pulsation in the region of the heart, and a mirror placed over the face presented no sign of respiration. Applying the instrument to the female, she trembled and gnashed her teeth and pouted her lips, which so frightened me that I resolved never to operate again without being quite sure." Pere then evidently acknowledges that he had determined too soon, to perform the cæsarean operation, and the fault which he committed cannot be attributed to art.

Finally, it is said that the Abbe Provoost, struck with apoplexy, in the forest of Chantilly, was considered dead, and that a surgeon of the village opening the abdomen, by the direction of the public officer, with the view of finding the cause of death, the celebrated author of Masson Lescant cried out and died in a few minutes.

This anecdote has been received as true by many legal physicians, and by the authors of the Biographie Universelle. Dr. Bouchut, after extensive investigation, is convinced that there is no foundation for the belief that this horrible accident had occurred. For the rest, on the other hand, the whole blame rests with the surgeon, for it is well known in apoplexy, that the circulation continues and the pulse may be easily felt.

The author of the memoir concludes his remarks on these cases by saying, that of the three most serious errors with which the physicians are charged, two were probably not committed, and not one can fairly be attributed to the uncertainty of the science.

The second category of facts relative to apparent deaths taken for actual, and followed by premature inhumation, comprehends a number of cases which must unhappily be considered as too true. The authors of these mistakes were parents, servants, nurses or other persons who had not sufficient intelligence to distinguish between life and death. Several individuals which they have considered dead, and were preparing to bury, have been saved by physicians, who, having opposed their burial, have succeeded in resuscitating life considered as lost.

Finally, a third category embraces a large number of histories, or anecdotes, whose details are unworthy of credit; and which have not even been admitted as true by most of the authors who have written on this subject.

With the impossibility of now deciding on cases published long ago, the author has examined and discussed those of more recent occurrence, and shows by public documents, obtained from the vicinity by means of the local public authorities, the uncertainty and falsity of these accounts. In fine, persons unacquainted with medicine after a too hasty and superficial examination, have pronounced persons dead who were not. These unhappy mistakes have always been made by individuals unacquainted with physiology or medicine, and the number of cases has been exaggerated.

The author then takes up the first question proposed by the Academy. *What are the characteristics of apparent death?*

The observations and experience of Dr. Bouchut have brought him to this result, viz: that all the apparent deaths, especially those resulting from asphyxia and syncopy, present, whatever may be the diversity of the symptoms, a common character, *the continuation of the pulsations of the heart,* which distinguishes them from those of real death.

This important fact, in the history of these cases, has attracted the particular attention of your commissioners. Not only have they repeated the observations of Dr. Bouchut, on the persistence of the action of the heart in cases of apparent death, but they have gone so far as to make new experiments in order to give this character its full value.

Since the time of Frederick Hoffman, syncopy has been generally attributed to a complete cessation of the functions of the heart. This opinion was professed by Bichat and his followers in France, and has been repeated by the more recent medico-legal writers. Dr. Bouchut has stated, that in the most complete state of syncopy, with loss of sensation and motion, and with coldness of the body, there is not an entire cessation of the contractions of the heart, but only a diminution in the frequency and force of its action. To the support of this opinion, Dr. Bouchut cites the case of a man, who, after a wound of the radial artery, has so considerable a hemorrhage, as to induce, in a short time, several severe syncopies. Insensible to stimulants, he was completely unconscious; his body as white as marble, was cold; no pulse in the wrist; the beating of the heart imperceptible to the hand; but by auscultation it could be merely perceived at distant intervals.

In other analagous cases, characterised by a general palor, coldness of the body, loss of intelligence, of sensibility, and of movement; cases in which respiration was imperceptible or very weak, the author states that the pulsations of the heart, more or less feeble, were reduced to twenty or even fifteen per minute. But, in all these cases of syncopy from hemorrhage carried to the greatest extent, these beatings of the heart were easily perceived by auscultation, and thus distinguish apparent from real death.

Not only in cases of syncopy, the effect of extensive hemorrhage, does Dr. Bouchut insist on the existence of pulsations of the heart; for he cites the case of a young hysterical woman who became suddenly motionless and voiceless, whose limbs were completely rigid, the surface and senses entirely insensible, in whom the action of the heart proved that life was not extinct.

Two of the commission (Magendie and Rayer) have several times observed the pulsations of the heart in very severe syncopies, which presented all the appearance of cases of *apparent death.* James Copland and M. Piorry have also expressed the opinion that the action of the heart continues in most, if not in all cases of syncopy; but the commission is of the opinion that no one has been so accurate, or demonstrates the importance of this fact by so many cases as Dr. Bouchut.

While, however, we rarely observe syncopy in men, and as cases of syncopy are of unequal severity, and as some are so slight as not to be considered cases of apparent death, two of the commission (Magendie and Rayer) have thought that they ought to test the value of this sign (the pulsations of the heart) by numerous experiments on animals which approach man in the character of their organization.

In these experiments, your commissioners have produced syncopy in every degree, frequently almost to death, and to death itself. Without entering into a long detail of these matters, we will confine ourselves to a statement of the means employed, and the facts observed. After having adjusted a syringe to the carotid of an animal, a quantity of arterial blood was drawn. This was repeated (the emission) several times until the animal was in a condition apparently dead, sometimes so decidedly that when the syringe was taken from the carotid, this vessel not only did not pulsate, but scarcely bled, or did not at all. In this condition, the animal entirely insensible, evacuated its urine and fcæes as animals do in articulo mortis; the gums and inside of the lips were pale and cold; the limbs contracted: the cornea and globe of the eye insensible to the touch, and respiration very little or not at all apparent. Now in this state of syncopy, the pulsations of the heart were easily perceived by auscultation, only the two sounds were not perceptible, but were sometimes represented by a simple *tac,* which was however very distinct.

A large abstraction of venous blood produced the same results. In order to determine the symptoms of apparent death, in animals, by the abstraction of venous blood, it is not sufficient to open the large veins, it is necessary to introduce a tube into the right side of the heart, drawing the blood out by means of a syringe, taking care not to allow of the ingress of blood, to the heart. One of these animals, after the loss of venous blood, was thrown into so complete a state of syncopy, that the cornea was insensible to the touch; the pupil at first contracted as in the ordinary agony, became expanded as in the instant of death. In this animal, entirely insensible and motionless, although its cords were removed, and it was excited by being pricked, for some time occasional pulsations of the heart were observed. Two minutes after the last pulsation of the heart, on opening the chest, the auricles presented a vermicular movement, similar to that observed when the heart is removed from an animal.

In some of our experiments on apparent death, produced by the abstraction of blood from the right cavities of the heart, a quantity of air having been introduced into its cavities, the pulsations of this organ were not only very distinct, but were accompanied with a true gurgling, (gargouillement) and the animal died immediately.

To sum up, our observations on man and expériments on animals, experiments in which syncopy was carried to the greatest extent, have fully confirmed the fact, on which the author of the memoir has insisted, viz: the persistence of the pulsations of the heart in syncopy, and their perception by auscultation.

We proceed to add a few remarks in reference to certain syncopies, independent of hemorrhage, which are said to have been observed in persons who had the singular faculty of voluntarily affecting the actions of the heart. The following fact (particularly in works on medical jurisprudence) has been frequently quoted; Col. Townshend, long an invalid, called Drs. Cheyne and Baynard, and Shrine his apothecary, to witness the singular experiment of dying and coming to life again. The Colonel placed himself upon his back; Dr. Cheyne applied his fingers to the radial artery, Baynard applied his hand to the region of his heart, and Shrine held a mirror to his mouth. In a moment he appeared dead, respiration ceased, with the pulsations of the arteries and heart. The looking-glass was not tarnished; a half an hour elapsed, and the spectators were about to retire, persuaded that he had become a victim to his experiments, when they perceived a slight respiratory movement, the pulse returned by degrees, and the invalid became conscious. He then sent for his notary, added a codicil to his will, and died very quietly eight hours afterwards.

Dr. Bouchut remarks, with justice, that all this has the air of improbability. But supposing that it be true, it does not prove that the heart did not beat in the first part of the experiment; for auscultation has shewn, latterly, the continuation of the pulsations of the heart in large numbers of cases when the pulsations of the radial artery had entirely ceased, and in which the beating of the heart was not perceptible to the hand. All the physicians say that was the case very often in the Asiatic Cholera, in Paris, in 1832.

The other examples of voluntary and complete suspension of the circulation and respiration, cited by Haller, are not more conclusive or reliable.

We will close these observations on the state of the heart in syncopy, with apparent death, with a remark which illustrates the importance of auscultation in the prœcordial region. The following case has often been cited. A pregnant female was considered dead for two hours. Regandeaux examined her, and could perceive neither the pulsations of the heart, nor those of the arteries. The mouth was frothy, the abdomen much enlarged, the orifice of the uterus well dilated, filled with the bag of waters. Rigaudeaux decided to turn the child and deliver by the feet, thinking it dead; with care and attention it was resuscitated in about three hours. Examined a second time by Rigaudeaux seven hours after, she was considered dead, she presented no sign of life; but as the limbs were not cold, he forbade her burial, and two hours and a half afterwards he was told that she was restored to life. At present in a case like this, the physician need not be so long in doubt; by attentively auscultating the hearts of the fœtus and of the mother, the proof of life could be obtained. And a surgeon called to a case even more serious than that, viz: to a parturient woman, expiring, auscultation will be the means of detecting the continuance or cessation of the pulsations, and decide the moment when it is proper to hasten the delivery of the living infant, the mother being dead.

In asphyxia from strangulation, with apparent death, auscultation must equally decide as to the continuance of life. This conclusion results not only from clinical observations, but from experiments made upon animals. In this experiment the beating of the heart became slower, and slower; from 350 in the rabbit, it falls to 40 per minute, a convulsion occurs, and when the immobility and insensibility of the animal, with the absence of respiratory movements, seem to indicate that death has taken place, the movements of the heart are yet for an instant perceptible by auscultation. But after a moment's silence or complete absence of pulsations, insufflation of the lungs will not restore them, the animal is dead.

To observe all the modifications which the beatings of the heart undergo in all the degrees of asphyxia, from the beginning up to the instant when, after appearing dead, the animal is really dead, two of your commissioners (Magendie and Rayer) repeated the following experiments a large number of times: They attached to the trachea of an animal, a tube with a faucet, which permitted the tube fo be closed or opened at will. They have stated, as did Bouchut, that in apparent death resulting from asphyxia, from deprivation of air, there was a diminution of the pulsations of the heart from 300, or from 200, which they are per minute, (according to the age and the degree of agitation of the animal) to 20; in this depressed condition of the action of the heart, real death follows apparent death in a period which never exceeds two minutes.

It is known that infants, after delivery, have sometimes remained motionless and voiceless, and without respiration or any other external sign of life. Now, in this state of apparent death, known by the name of asphyxia of new-born children, auscultation is the means by which the physician detects the action of the heart and life.

In the table list which has been made of apparent deaths produced by cold, especially cold in the summer season, several authors have mentioned the diminution, and even the absence of the pulse; but auscultation has not been resorted to in these cases.

Dr. Bouchut cites the case of a man found on a public highway, in the winter of 1843, who was taken in a state of apparent death into the hospital Necker. The skin on the limbs was cold, covered with livid spots. There was no sign of sensibility or intelligence; the limbs were completely rigid, respiration almost imperceptible; yet, by means of auscultation the heart was observed to beat 32 times per minute.

In submitting animals to the influence of refrigerating mixtures, Dr. Bouchut observed that the decrease in the heart's action may be much greater. The following is his report of one of these experiments:

Twenty minutes past 12 o'clock, a rabbit was placed in a mixture of ice and salt. The animal had 25 to 30 pulsations of the heart in five seconds, or 300 to 350 arterial pulsations per minute. In a short time it appeared to sink; at one hour there was difficulty of respiration, 140 pulsations; in an hour and five minutes, 100 pulsations; in an hour and ten minutes, 40 pulsations; general convulsions of the trunk and extremities during three minutes; in an hour and a quarter, for four or five minutes the action of the heart could be perceived. In one hour and twenty minutes, the pulsations of the heart having ceased, pulmonary insufflation did not restore the animal to life.

Your commission have also produced apparent death in animals, by submitting them to the influence, more or less active, of frigorific mixtures, and have observed the progressive diminution of the heart's action. In several cases, where the trunk and members were really frozen, and solidified by cold, they have seen the beating of the heart reduced to four per minute. Besides this, they have made an operation well calculated to demonstrate the action of the heart in the last moments of life. In frozen animals, whose corneæ were insensible and flattened, having exposed the precordial region, a platina needle, passed into the heart, indicated, by its oscillations, the beating of the heart from 10 to 8 times per minute. One of us, with the assistance of the stethoscope applied to the chest of the animal, could perceive the pulsations of the heart within, to correspond with the oscillations of the needle without.

M. Weber has shown that the movements of the heart in a frog may be suspended for two minutes, by applying to this organ the extremeties of two threads of an electro-magnetic apparatus, and that the heart may afterwards resume its natural movements. They have also shown that when the poles of an electro-magnetic machine are applied to the medulla oblongata, or to the separated origins of the par vagum nerves of a frog, that the movements of the heart which fill it with blood are suspended. Two of your commission (Magendie and Rayer) have repeated these experiments also: they have observed, besides, that if two needles are passed into the chest of animals which are the nearest to man in their organization, such as a rabbit, a 'cabiais,' or a dog, &c, and place these needles in contact with the two poles of a pile, the movements of the heart will be suspended, but for a few seconds only. Your commission desire to recall their experiments to the physicians whose duty it is to examine the condition of the heart in these, fortunately very rare cases of apparent death from lightening, in which the suspension of the movements of the heart may be more decided than in ordinary cases, without being confounded with a final cessation.

It is known that some poisons act so energetically and promptly, that apparent death takes place immediately, to give place in a few moments to real death. The author of the memoir having made no experiments on this subject, your commission have thought it necessary, on account of the importance of the question, to examine for themselves into the condition of the heart in these circumstances. The results which they have arrived at, in all these experiments, fully confirm the law of the persistence of the pulsations of the heart, when death is but apparent.

They proceed to state a few of their experiments:—A rabbit was submitted to the action of a powerful poison. A grain of curare was placed under the skin, at the fold of the groin; six minutes afterwards the animal was in a state of apparent death; the pulsations of the heart fell from 220 to 72 per minute; very soon it was less and less frequent, then it could no longer be perceived. Two minutes of their cessation, the animal was opened; the ventricles of the heart were immovable, the auricles alone presented slight vermicular contractions.

Your commission would also state, that by the action of alcohol or of the preparations of digitatis, they could not produce apparent death, without the persistence of the beatings of the heart or contractions of the ventricles, perceptible by auscultation. The tenth of a grain of digitaline dissolved in alcohol, was introduced into the subcutaneous tissue of the back of a dog of middle size. At the end of five minutes, no effect being observed, another tenth of a grain of digitaline, dissolved in about six grains of alcohol, was gently injected into the jugular vein. Before the end of the injection, the beatings of the heart, which one of us listened to attentively with the stethoscope, ceased instantly; they were no longer obvious by auscultation, nor indicated by a needle passed into the heart, through the chest. After a half minute's suspension of the pulsations, a beating was perceived; afterwards the number perceived by auscultation, rose to 8 per minute, then to 12 (a number which always corresponded with the oscillations of the needle). The animal vomited, had convulsions, and the pulsations of the heart ceased, no more to return; the animal was dead; the heart was greatly distended and filled with black blood, the tissue of the heart, and even that of the auricles, did not contract when excited by the point of a needle.

In another experiment, the injection of digitaline dissolved in alcohol instantly arrested the action of the heart; for three minutes the needle introduced into the heart indicated no contraction, the ear perceived no sound. The animal was dead. Six grains of alcohol injected into the heart of a rabbit by the jugular vein, arrested almost instantly the heart's action; the ear and the needle denoted no contraction for two minutes, the animal was dead; on opening the chest the heart was irritated with the point of a scalpel without producing contractions; the cavities of the heart were filled with black blood. In other experiments with these means, the same results were obtained, viz: death more or less rapidly indicated by the cessation of the cardiac sounds.

Formerly, examples of apparent death were confounded with certain cerebral affections, which were accompanied with loss of sensation and motion. Dr. Bouchut thinks, with reason, that the Academy did not demand a description of all these diseases, nor the comatose or lethargic conditions which many of them present. He has confined himself to the characters which distinguish real death. In all thesecases, as in the soporose conditions produced by narcotic poisons, the condition produced by prussic acid, the insensibility produced by chloroform, the existence of life is determined by auscultation. Dr. Bouchut remarks that observations made upon the sleep of hybernating animals, present considerable interest in reference to this subject. In the waking state, the warmest has 90 pulsations; in the sleeping and numb condition, the number is reduced to 8 or 10 per minute. Here the cardiac action, as in all other cases, is the evidence of life.

To sum up, apoplexy, epileptic or hysteric coma, narcotic poisons, diffusible poisons, alcohol, ether, chloroform prussic acid, &c.; congestion, asphyxia and syncopy, in all their forms and degrees; all the cases, in fact, which are cited as examples of apparent death, may be distinguished from real death by the pulsations of the heart.

Such is the answer made by the author to the first question proposed by the Academy, and it appears to us descisive.

The second question propounded by the Academy is as follows:

*What are the means of preventing premature burials?* The present legislation in reference to the deceased is insufficient.

In directing the public officer to publish the death, and requiring to allow twenty-four hours to elapse between the publication and the burial, the authorities consider that they have taken all necessary measures for preventing premature burials; but it must be remembered that the mere dictum of the officer of the civil authority does not present all the guaranties desirable.

The municipal ordinances require the physicians to state the deaths, in the large cities. This wise precaution will in future become a dead letter in the law.

The author of the memoir thinks, very properly, that this measure should be established all over France, as well in the small as in the large towns, in the country as well as the city. It is in vain to object that the large cities only can bear the expense necessary in these cases; that a large proportion of the country districts could not support this new charge; the measure should apply to those cases which cannot be postponed. It is to the signs of death that we are to look for protection against the dangers of being buried alive.

According to Dr. Bouchut the signs of death are either present and certain, or distant. The certain signs of death in man, are:

1st. Prolonged absence of the pulsations of the heart, by auscultation.

2nd. The simultaneous relaxation of all the sphincters, due to the paralysis of the muscles.

Finally the flattening of the globe of the eye and opacity of the cornea.

In the opinion of your commissioners, each of these signs has not equal value, or certainty. Some remarks on this subject are necessary.

Since the admirable discovery of Laennec, we look in vain in science, for a single fact or a single rigorous experiment, to establish in man the continuance of life after the prolonged cessation of the pulsations of the heart as detected by auscultation; yet it is necessary to bear in mind how far the diminution of the heart's action, with a suspension of longer or shorter duration, may exist without a final cessation.

The expression of prolonged absence, employed by the author of the memoir, to indicate the definite cessation of the heart's action, does not appear to your commissioners sufficiently accurate, practical or precise. They have thought that it was necessary to fix a limit which would leave no doubt on the reality of the final cessation of the functions of the heart.

The study of the pulsations of the heart in a great number of cases of death, would furnish useful data for this decision. It is true that in the last agony, the sounds of the heart are often marked by a noisy râle, which obscures their perception; but in the intervals which separate the last inspirations, and always in the last moment when the râle begins to cease, the last beatings of the heart may be perceived, by applying the ear to the cardiac region. In this silence, so near death, they are very distinct, long even after the hand applied to the chest distinguishes no movement; and the arterial pulsations, in the neck or extremities, are no longer perceptible. Now, in this state, and especially in the silence which follows the last expiration, the maximum interval between the pulsations of the heart, has appeared to Dr. Bouchut, for an adult man, about six seconds. The observation of numerous agonies of death, has afforded your commissioner (Rayer) nearly the same result, that is to say about seven seconds, as the maximum interval between the last beats of the heart.

After these clinical observations, your commission think that the absence of the pulsations of the heart, perceived by auscultation, over all the points that they may be either naturally or accidentally detected on each, *during an interval of five seconds*, that is to say during a space of time fifty times longer than that which is given by observation on the sounds of the heart, in the last agonies of death, will leave no doubt of the final cessation of the movements of the heart and on the reality of death.

Otherwise the final cessation of the beatings of the heart is always accompanied with two very striking phenomena, and easily established; viz: the cessation of the respiratory movements, and the loss of sensation and motion. We may conclude, then, *that death is certain when we observe, in man, the definite cessation of the heart's action, which is immediately followed, when it has not been preceded by, the cessation of the functions of respiration, and those of sensation and motion.*

It has been objected, it is true, that the proofs of the cessation of the beatings of the heart, and of the cessation of the circulation, were insufficient, at least in certain cases; and that many persons in whom the cessation of the circulation was supposed to exist, have been restored to life. The objection was made, and the error was committed at a time when we could not, by feeling the pulse, and applying the hand to the region of the heart, decide with certainty on the state of the circulation. But the authors who, at this day, bring forward this objection, forget that by means of the ear, their pulsations may be detected, when neither the pulse or the hand applied to the chest, will indicate their existence.

The definite cessation of these pulsations is indicated by the cessation of the sounds of the heart. The observations of both the author and the commission leave no doubt in this matter. At the same time there is a fact which we desire to recall. A learned physician in Dublin, Dr. Stokes, says that in the typhus fever of Ireland, a disease in that unhappy country, that he observed not only a great feebleness of pulse with no perception of the heart's action, by the hand applied to the chest; but a very decided diminution in the first sound of the heart, and sometimes even the entire absence of this sound, which it is known is naturally duller and less distinct than the second. But in no case has he observed the entire absence of the sounds of the heart before death, in individuals attacked with a disease which affects so decidedly the circulation.

It has also been objected, that a considerable collection of serosity in the pericardium, or an emphysema of the lower part of the left lung, may entirely obstruct the perception of the beatings of the heart by auscultation; but this assertion is not well founded. In these circumstances it will not deceive the ear of the physician, whose examination should never be finished while observing any flatness to any considerable extent, or an unusual sonorousness in rhe region of the heart. We will add (for, in a case as serious as that of deciding on death, we should not fear to mention the most casual incidents) that auscultation, in cases of apparent or real death, should be resorted to over the whole chest, and both sides of the body, as the heart may be misplaced or pushed to the right side, in the rare cases of transposition of the viscera.

The physician should also distinguish simple suspension of the respiration from its final cessation. In the absence of rigorous observations on the maximum duration of the suspension of respiration in man, we will find a definite proof of the final cessation of respiration, in the fact of its coincidence with the phenomena of the final cessation of the heart's action. Up to this time, this sign has not been insisted on being taken from the state of the heart, a sign which indicates with certainty that respiration has ceased forever.

Many of the means taken to judge of the absence of respiration are completely at fault. It is supposed that respiration has ceased, when the flame of a taper placed before the mouth and nostrils, remains unaffected. Yet it is known that in gentle and feeble respiration, there is no appreciable movement of these bodies. On the other hand, the humidity on a looking-glass, applied to the mouth, is considered a sign of respiration; but this surface may be tarnished by the vapour which exhales from a corpse which is not yet cold—or from the humidity of the air. Another experiment is much more certain. In observing attentively the chest, and the abdomen divested of all covering, the complete immobility of these cavities with absence of the respiratory murmur on auscultation, indicate the absence of respiration; the persistence of the pulsations of the heart, will indicate that this function is only snspended; the cessation of these pulsations announce that respiration has ceased for ever.

To the final cessation of the heart's action, also, must we refer, to decide, with certainty, on the abolition or suspension of the functions of the nervous system.

To judge of the condition of life or death, by the condition of the nervous system, it is proposed to titillate the nostrils, to apply irritants to the schneiderian membrane, such as ammonia, acetic acid, &c; to resort to vesicatories, fire with a hot iron or hot oil, to incisions, to pinching the nipple, &c.; but these means produce neither sensation nor motion in individuals affected with serious cerebral affections, and still less those under the influence of chloroform or ether. The complete loss of sensation and motion is compatible with life, but when the action of the heart has ceased, it becomes the most striking evidence of death.

Your commission, then, think with the author of the memoir, that the definite cessation of the action of the heart and arteries, known by auscultation, is a true sign of death; a sign the more certain, inasmuch as the cessation of the heart's action is followed, if not preceded by, the cessation respiration and the functions of the nervous system.

The second immediate sign of death, admitted by Dr. Bouchut, is not in the opinion of your commissioners sufficiently certain, This sign is the simultaneous relaxation of all the sphincters from paralysis. According to the author, many of these muscles may be paralyzed during life; but he has never observed the simultaneous relaxation of all these muscles during life.

Your commissioners know that the sudden and almost instantaneous relaxation of all the sphincters, including that of the pupil, is in man, in a great majority of cases, the effect of death and not of disease. At the same time, we would not affirm that the general paralysis of the sphincters cannot exist in man previous to death. The relaxation of all the sphincters takes place in many of the agonies, when ausculation reveals the pulsations of the heart; and certain central affections may induce at once the relaxation of the sphincters, with that of the iris. The fact of this relaxation in the dying, cannot be observed except by a physician accidently or intentionally stationed near the individual; and cannot be observed in a large number of cases. It is certain also, that we cannot in some minutes, by dividing the optic, the two seventh pairs, and the spinal marrow in the dorsal region, producing paralysis of the pupil, and the other sphincters, without producing immediate death. Two of your commissioners (Magendie and Rayer) have also verified, on decapitate animals, by prolonging, artificially, respiration, the fact that the beatings of the heart, in animals thus mutilated, remained very distinct and regular during several minutes.

From all these considerations, your commissioners think that the second sign of immediate death, admitted by Dr. Bouchut, is not sufficiently certain.

A third sign of death, considered as certain by Dr. Bouchut, appears to us of no greater reliability than this. Dr. Bouchut thinks, with the celebrated author of Letters on the Certainity of Death, that the formation of a glazed veil on the surface of the cornea, with flattening of the globe of the eye, is a certain sign of death. In opposition to this assertion, we have observed these symptoms in the Asiatic cholera, several hours before death, while the pulsations of the heart were yet perceptible by auscultation, and when the arterial pulsations were really imperceptible.

To conclude, of the three signs of death admitted by Dr. Bouchut, there is but one, whose certainty is admitted by us. In pointing out a sign so positive, and generally so easily verified, to the attention of the profession, Dr. Bouchut has filled up a hiatus, left open by the authors of legal medicine, among the signs of immediate death. In reference to the *proximate* signs of certain death, Dr. Bouchut admits three, viz: cadaveric rigidity, the absence of muscular action under galvanic agents, and putrefaction.

The certainty of these signs is admitted by all medico-legal writers, and cannot be denied, so positive are the observations and experiments on which they are founded. In this part of his work, the author has explained with care the condition of the science, and refuted some objections which have been advanced, by the partizans of the dead houses.

For a long time, formerly, cadaveric rigidity was regarded as a sign of death; but the demonstation of the importance and certainty of this sign is due to Louis and Nysteu, two French physicians. After death, the flexibility of the limbs disappears; the muscular tisue hardens; the limbs become immovable and stiff. No convulsive or tetanic condition presents this succession of phenomena, so as to deceive a physician. In cadaveric rigidity, the limbs yield to attempts at extension or flexion, as they do in an inanimate body. In convulsive affections the circulation continues; in cadaveric rigidity the pulsations of the heart, respiration, and the central functions, have entirely ceased.

The entire absence of the phenomena of muscular irritability, under the influence of different excitants, and of galvanism, is also a certain sign of death.

This fact has also recently been tested. It is pretended that in asphyxia produced by carbonic acid, and especially that produced by sulphuretted hydrogen, the muscles sometimes give no evidence of muscular contractility, by galvanism, before death. To test this matter, two of us (Magendie and Rayer) have made the following experiments:—A rabbit was asphyxiated by carbonic acid gas. After being accelerated, respiration diminished, and the animal fell, without convulsions, into a condition of apparent death, characterized by complete immobility and insensibility, the absence of respiratory movements, and by a considerable diminution in the actions of the heart, as determined by ascultation. A muscle of the thigh was placed in connection with one pole of a battery, while the other pole was placed in connection with the neck, which produced several shocks. This experiment was repeated several times, with the same result. For a time the pulsations of the heart were perceptible, they then ceased. A needle pushed into the heart through the intercostal muscles, presented no oscillations.

The chest opened, the heart was found motionless; the animal was dead. The muscles still contracted as often as the electricity was applied.

In another experiment, a dog of medium size and vigorous, was asphyxicated by sulphuretted hydrogen; under the influence of this energetic poison the animal was soon much agitated, it then fell into a state of apparent death, characterized by immobility and insensibility so complete, that no movement or appearance of feeling could be induced by the most violent pinching of the lips and tail. The respiratory movements were suspended; the beatings of the heart very slow, but very distinct, alone indicated life. A muscle of the thigh subjected to the influence of electricity, contracted powerfully for an instant after the cessation of the heart's action. The chest being immediately opened, the heart was found immoveable. After death the muscles of the limbs continued to manifest contractions on the application of electricity. Muscular irritability, then, is not destroyed in asphyxia from carbonic acid, nor from sulphuretted hydrogen. The abolition of this property of the muscular fibre remains a certain sign of death.

But this sign may not be appreciable until after a considerable time; the functions of the heart, of the lungs, and of the nervous system, may have ceased for a long time, death may have taken place, and the muscles will still contract on the application of various stimulants. Dr. Bouchut has referred to the observations which demonstrate the value of the phenomena of putrefaction or of cadaveric decomposition, considered as a sign of death. Death may always be distinguished long before the establishment of decomposition. The details into which Dr. Bouchut has entered on this matter appears to us to be justified by the efforts lately made in France, to create mortuary houses where bodies may be deposited until decomposition.

It is known that at the beginning of this century Hufeland, and several other physicians, having maintained that all the signs of death were uncertain except putrefaction, mortuary houses were established in many towns in Germany; at Frankfort on the Maine, where there was the most noted one, Hamburg, Wisbaden, at Weimar, &c. But although most of these still exist, yet their utility has become doubtful. The most of them are badly conducted, and their interior organization is very imperfect. Finally, for fifty years that these houses have been established, no body which has been placed within them, after a certificate of the physician had been given, has come to life.

To create this day, in France, dead houses to contain bodies until putrefaction, is to engage in an endless expense, which a large proportion of the towns and districts cannot afford; but it is taking no notice of the other certain signs of death.

These remarks are not designed to apply to the creation of desirable places to receive, shortly after death, the bodies of the poor, whose families have not room for them.

It has been lately announced as a certain sign of death, the impossibility of raising bullae or blisters on the skin of the dead, by the application of boiling water or other excitants. Dr. Bouchut says this sign is uncertain: he has, indeed, produced by the action of heat or boiling water true blisters on the dependent or infiltrated parts of several bodies, some of which had begun to putrify.

It is true that blisters produced by hot water, during life, are generally surrounded by a red circle which those in the dead body are not; but Dr. Bouchut has seen old men and cachectic individuals (four hours before the cessation of the beatings of the heart) with the skin burned, presenting no red circles around the burnt points. One of your commissioners (Rayer,) in burning persons in the last agony, with the hope of restoring them to life, or of prolonging life, and doing the same on dead bodies, has arrived at pretty much the same conclusion as Dr. Bouchut. Indeed the areola which is formed, commonly, around a blister caused by a burn, is scarcely, or not at all visible in persons of color. The development of a blister and the deposit of a quantity of serosity under the epidermis, after the application of boiling water, cannot then be considered as a certain sign of life, sufficient to distinguish it from death. To condense this second part of Dr. Bouchut's work and the facts contained in it, your commisson present:

1*st.* That the definite cessation of the pulsations of the heart, indicated by the cardiac sounds, is an immediate and certain sign of death.

2*d.* That cadaveric rigidity is equally a certain sign of death.

3*d.* That the absence of muscular contractions under the influence of electricity is a third certain sign of death.

4*th.* That general putrefaction of the body does not generally take place until long after the preceding conditions; that it is not necessary to wait for decomposition before proceeding to bury or embalm.

5*th.* That the cessations of the pulsations of the heart and of the circulation, the development of cadaveric rigidity, the abolition of the muscular contractility, cannot be appreciated except by physicians; the announcement of death should be confided exclusively to them, both in town and country.

6*th.* The posibility of establishing the existence of death previous to putefaction, renders the establishment of dead houses, such as are found in Germany, unnecessary; but that it is desirable that the bodies of the poor should be received until sepulture.

The importance of the questions proposed by the Academy, the manner in which Dr. Bouchut has studied them, and revolved them by new observations: the numerous experiments performed by your commissioners, will justify, we hope, the length of this report.

The work of Dr. Bouchut, excepting some imperfections, the most serious of which your commissioners have pointed out, appears to them remarkable for its terseness, the precision of its details, by the judicious manner in which the facts in relation to apparent deaths have been appreciated, by the profound discussion of the observations which have been made relative to cadaveric rigidity, the abolition of contractility, considered as certain signs of death, and, above all, the care which the author has taken to demonstrate that the *continuance* of the pulsations of the heart is the distinctive characteristics of apparent deaths, and that the cessation of the pulsations of this organ, detected by auscultation, constitutes an immediate and certain sign of death; a primary fact by which the author has responded to the call of the Academy, which demanded, especially, all necessary aids in rendering the distinction between apparent and real death more prompt and more sure.

WIth these considerations, your commissioners have unanimously awarded the Prize of Manni to Dr. Bouchut, as the author of the best Memoir addressed to it during ten years, that is to say since 1837, the time when the announcement of the prize was first made.

The conlusions of the report were adopted.

